# A two-year dataset of georeferenced unproductive vines in a southern France Mediterranean vineyard

**DOI:** 10.1016/j.dib.2026.112626

**Published:** 2026-02-26

**Authors:** Yoann Valloo, Romain Girardot, Pauline Faure, Bruno Tisseyre

**Affiliations:** ITAP, Univ. Montpellier, INRAE, Institut Agro, 2 Place Pierre Viala, 34060 Montpellier, France

**Keywords:** Grapes, Missing vines, Dead vines, Soil resistivity, Spatial data

## Abstract

This study describes a dataset of georeferenced unproductive vines (dead and missing) and associated observations on fields and soil. The dataset results from a survey of 14 287 unproductive vines performed in a French Mediterranean vineyard made of 50 blocks with a total area of 28 hectares. The survey was carried out over two successive years, 2022 and 2023. Geolocation-accurate data (<5 cm) was collected via a Real Time Kinematic (RTK) Global Navigation Satellite System (GNSS) and includes detailed information of the 50 vineyard blocks, such as boundaries, area, grape variety, plantation density and location of surveyed unproductive vines. In order to provide information on the soil situation, apparent soil resistivity data at three depths (0-50cm, 0-1m, 0-1.5m) acquired with Multi-depth continuous electrical profiling (MuCep) is also provided. This dataset has already been used to investigate optimised sampling methods aiming at assessing the proportion of unproductive vines at the field level. To our knowledge, a spatialized dataset of this precision and scale concerning unproductive vines is currently unavailable. This dataset is valuable for driving any experimentation aimed to study the spatial distribution of unproductive vines, potential correlations with soil characteristics, variety, and other factors. Furthermore, this data is relevant to consider the feasibility of estimating the proportion of unproductive vines, for example, via remote sensing techniques.

Specifications Table

**Table:** 

Subject	*Agricultural Sciences: Agronomy and Crop Science*
Specific subject area	Precision viticulture
Type of data	Spatial data files (consisting of .shp and associated files), Text table (.csv), Raw Data
Data collection	Unproductive vines data, with a distinction between missing vines and dead vines, were acquired in 2022 and 2023 over a 28-ha vineyard estate. They were geolocalized manually using a real-time kinematic (RTK) Global Navigation Satellite System (GNSS) receiver with an accuracy of 1 to 5 cm. Blocks boundary were also measured with the same RTK GNSS receiver. Blocks boundary correspond to the usual contour used in France to declare vines planted areas. For each block, information of the planted variety, area, date of plantation, etc. is provided. Soil apparent resistivity was acquired in 2017 using a Multi-depth continuous electrical profiling (MuCep) at three depths (0-50cm, 0-1m, 0-1.5m). Data of Soil apparent Resistivity was acquired and processed by a service provider (Geocarta, France).
Data source location	Institution: Institut Agro Montpellier City: Montpellier Country: France Data centroid coordinates: Latitude 43.5359010, Longitude 3.8534145 (EPSG:4326 – WGS 84)
Data accessibility	Repository name: Zenodo Data identification number: 10.5281/zenodo.17347615 Direct URL to data: https://doi.org/10.5281/zenodo.17347615
Related research article	[1] Oger, B., Laurent, C., Vismara, P., & Tisseyre, B. (2025). Towards estimating the proportion of dead and missing vines at the field level. *OENO One, 59*(1). https://doi.org/10.20870/oeno-one.2025.59.1.8061

## 1 Value of the Data

The data presented in this paper are an exhaustive count and location of 14 291 unproductive vines (4635 dead and 9656 missing plants – data of 2022) over a whole vineyard estate of 28 ha, made of 50 blocks. Due to the amount of work required, the acquisition of this data with a such spatial accuracy over two different years and more than 29 different grape varieties is rarely, if ever, carried out. The database also provides soil data (Soil apparent resistivity) measured across the entire estate with high resolution (1.5m) These data can be useful:

• To support or deepen any agronomic, ecophysiological, or other study investigating grapevine mortality [2]. This dataset could, for example, help identify biotic or abiotic factors (disease, environmental constraints, nutrients lack, etc.) that may explain mortality and its heterogeneity at different scales within blocks, between blocks and at the farm level. Given the diversity of grape varieties included in the database, the data can contribute to any scientific research aimed at studying the sensitivity of grape varieties to mortality.
• to advance research in remote sensing to design and test methods aiming at identifying, counting and locating dead and missing vines [3]. The dataset could, for example, be used to characterise the spatial, spectral or temporal resolutions needed to map unproductive vines.
• for analysing the spatial distribution of vine mortality and its potential links to available auxiliary environmental variables like soil, as well as auxiliary variables potentially accessible on dedicated platforms such as topography or over data derived from satellite imagery for example [2].
• to quantify and qualify sampling methods aiming at assessing unproductive vines at the block level in order to verify their effectiveness or to test new approaches driven by spatial ancillary data [1,4].

## 2 Background

These data were collected as part of the Mas numérique project carried out at l’Institut Agro Montpellier (France). They were originally collected and used to assess the effectiveness of commercial services specialising in mapping unproductive vines [5]. At least two research papers have used this dataset: i) to estimate the factors impacting estimation errors of missing plan when performed by human operator in order to provide guidelines for an optimal sampling plan [1]; ii) to correct the yield measured by a yield monitoring system during mechanical grape harvesting, in order to produce more relevant yield indicators, expressed as kilograms of grapes produced per productive vine stock [6]. Note that yield data and quality parameters data are also available over the whole vineyard estate with a medium spatial resolution of 0.3 ha [7] and with a very high resolution (∼ 2 m.) over two blocks of the same vineyard [8].

## 3 Data Description

**Fig. 1:**
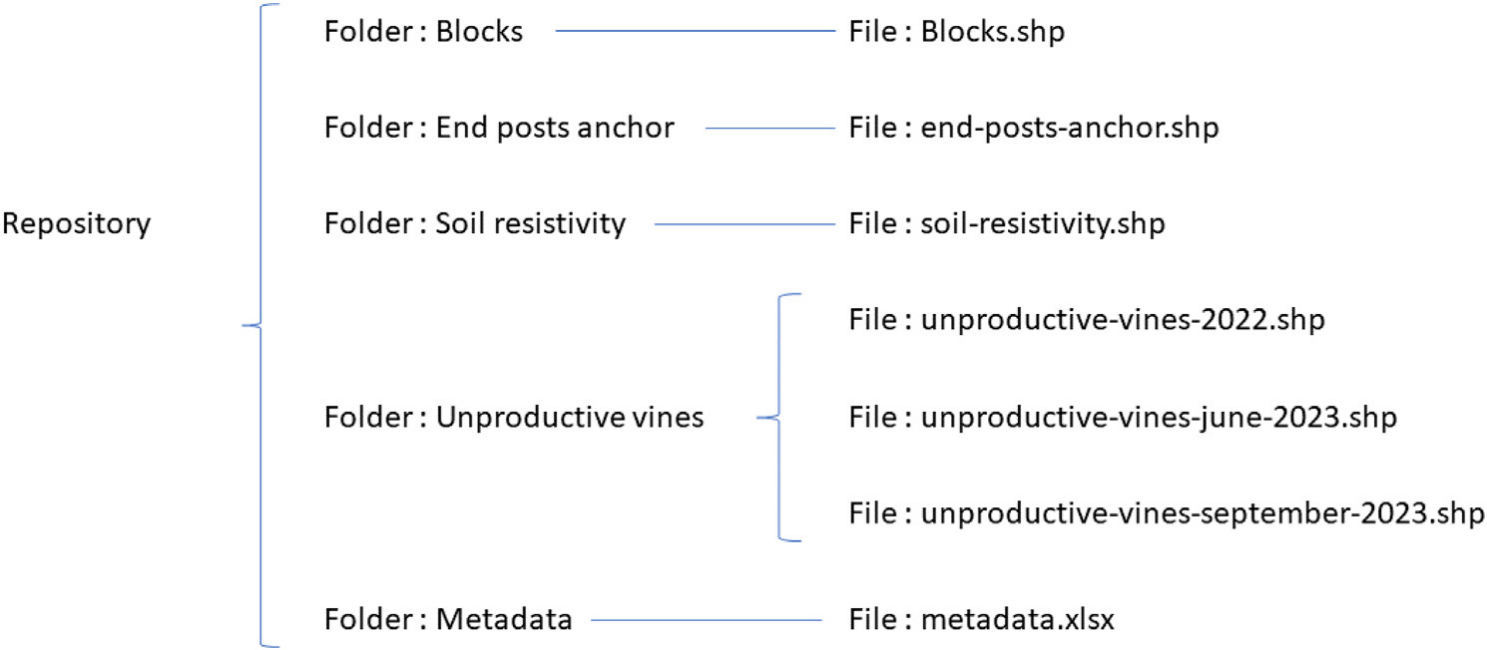
Data folder structure.

The organization of the dataset is presented in Fig. 1.

The data were obtained on a 28-ha vineyard estate located in the south of France (Villeneuve-lès-Maguelone, France; 43.532300° N, 3.864230° E). The vineyard estate is made of 50 blocks planted with 10 different grape varieties. The dataset includes 4 types of data:

- Blocks data: the data that describes every vine block of the vineyard. It includes the boundary of each block (spatial data) and for each block, its main characteristics like the grape variety, the name of the block, the inter-row distance, the number of rows, the inter-plant distance along the row, the planting date if available, the planting density and the area (Table 1). The data is provided as a geographical file (shapefile and associated files) using the RGF93 v1 / Lambert-93 system (EPSG 2154, the most precise system for France). There are 50 blocks in the dataset.

**Table 1:** Blocks data attributes.

Variable name	Description	Unit
Fid	Unique number for each block	
Variety	Variety of the block	
Name	Name of the block	
Interrow	Distance between each row for the block	m
Nb_row	Number of vine rows in the block	
Vine_spaci	Distance between each vine in the rows of the block	m
Plant_date	Plantation year if available	Year
Density	Number of vines per Ha	Number / Ha
Area	Area of the block	Ha

- End posts anchor (or moorings): locations of the end posts anchor of the vine rows. It's worth remembering that the vines are trained on a trellis system, necessitating the placement of anchors at both ends of the row to secure the trellising wires. The exact location of these anchors thus serves to delineate the beginning and end of each vine row. Each end post anchors of each row is then represented by a geolocated point in order to identify the vine row. The data is provided as a geographical file (shapefile and associated files) using the RGF93 v1 / Lambert-93 system (EPSG2154).
- Soil resistivity: the data that contains the soil resistivity measurements. This data was acquired by the company Geocarta [9] using a Multi-depth continuous electrical profiling (MuCep) [10] for measuring apparent soil resistivity at three depths (0-50 cm, 0-1 m, 0-1.5 m) pulled by an All-Terrain Vehicle (ATV) (Table 2). Each measurement is represented by a geolocated point containing the apparent soil resistivity measurements at three depths: one measurement between 0 and 50 cm (Depth50), one measurement between 0 and 100 cm (Depth100) and one measurement between 0 and 170 cm (Depth170). The data is provided as a geographical file (shapefile and associated files) using the RGF93 v1 / Lambert-93 system (EPSG2154). Soil resistivity data is available for 37 of the 50 blocks.

**Table 2:** Soil resistivity data attributes.

Variable name	Description	Unit
Fid	Unique number for each point	
Depth50	Resistivity to the passage of electric current in the soil over the soil layer from 0 to 50cm deep	Ω m (ohm-metre)
Depth100	Resistivity to the passage of electric current in the soil over the soil layer from 0 to 100cm deep	Ω m (ohm-metre)
Depth170	Resistivity to the passage of electric current in the soil over the soil layer from 0 to 170cm deep	Ω m (ohm-metre)
- Unproductive vines: the data that contain the geolocation of unproductive vines. There are two types of unproductive vines: dead vines, which are still present but no longer produce vegetation or grapes, and missing vines, represented by a gap along the row where a vine should be. Missing vines are often dead vines that have been pulled out. This dataset is composed of three files corresponding to three survey campaigns. The first file contains the unproductive vines in all the 50 blocks of the vineyard estate in 2022. It encompasses 14291 observations (4635 dead and 9656 missing vines). The other two files contain the geolocation of unproductive vines from the campaigns carried out in June 2023 (6665 observations) and September 2023 (7175 observations) on 17 of the estate's 50 blocks (Table 3). Each unproductive vine is represented by a geolocated point with information on the status of dead or missing plants. The data are provided as a geographical file (shapefile and associated files) using the RGF93 v1 / Lambert-93 system (EPSG2154)

**Table 3:** Unproductive vines data files and attributes.

File name	File Description	Variable name	Variable description
unproductive-vines-2022	Survey of unproductive vines throughout the estate in 2022	fid	Unique number for each point
unproductive-vines-june-2023	Surveys of unproductive vines on 17 of the estate's 50 blocks in June 2023	Status	Status of the unproductive vine: “Missing” if the vine is missing; “Dead” if the vine is present but dead
unproductive-vines-september-2023	Surveys of unproductive vines on 17 of the estate's 50 blocks in September 2023

## 4 Experimental Design, Materials and Methods

### 4.1 Experimental site

The experiment was conducted in 2022 and 2023 on a 28-hectare rain-fed vineyard located in Villeneuve-lès-Maguelone, southern France (43.532300° N, 3.864230° E - Fig. 3), at the commercial wine estate “Domaine du Chapitre.” The site is under Mediterranean climate. The vineyard consists of 50 individual blocks, all positioned within a 3 km radius of the estate’s central building. The soils across the site are predominantly comprising deep calcisols and cambisols with a silty, clayey, and sandy texture. These soils are highly calcareous (pH 7.9), moderately rich in organic matter (1.78%), and contain little gravel (<15%), contributing to a high capacity for water retention (>100 mm). The vineyard is managed using vertical shoot positioning with a three-level trellising system, and canopy management (trimming) is carried out during the vegetative stage, the first week of July. Vine density ranges from 3,300 to 4,000 plants per hectare depending on the block.

### 4.2 Dataset

Data on unproductive vines and on end posts anchor were collected manually by a trained operator using a smartphone, the Mergin Maps application (London, United Kingdom) was used to manage the project and store the collected data. An RTK rover was linked to the Mergin Maps application to geolocate points with an accuracy of 1 to 5 cm. The RTK rover was connected via Bluetooth to the smartphone, which received RTK corrections via GSM using the free Centipède RTK network [11]. The furthest block was no more than 2500 m from the RTK base station used to provide positioning corrections, these are ideal conditions for achieving high positioning accuracy of around 1 cm [12]. For the dead vines, the point was geolocated above the base of the vine. For missing vines, the point was geolocated by estimating its position hypothetically on the basis of the distance between surrounding vines and the known plantation density (i.e., the known distance between vines on the row).

Data collection from unproductive vines took place in three sessions. The first session of collecting unproductive vines took place in spring 2022. This session was carried out across the entire estate, covering all 50 blocks. The second and third collection sessions took place in June 2023 and September 2023. These two sessions were part of an experiment aimed at assessing the quality of digital services for estimating unproductive vines in vineyards. Only 17 of the estate's 50 blocks were involved in this experiment. Thus, for the both data collections in 2023, only 17 of the estate's 50 blocks were surveyed. When it is indicated that a block has been surveyed, this means that all the vines have been inspected to assess whether they are alive, missing or dead. Only missing and dead vines have been geolocated. This is therefore no sampling but a complete monitoring of all vines. For example, if the block was 1 hectare with a density of 4,000 plants per hectare, all 4,000 vines were inspected.

The boundaries of the blocks were acquired using the same RTK rover as the one used to geolocate the unproductive vines with the same methodology. The boundaries of the blocks correspond to the convex hull defined by the end-of-row anchors. Thus, only zones where vines are planted are included within these boundaries. Block headlands are, for example, excluded from the boundaries.

**Fig. 2:**
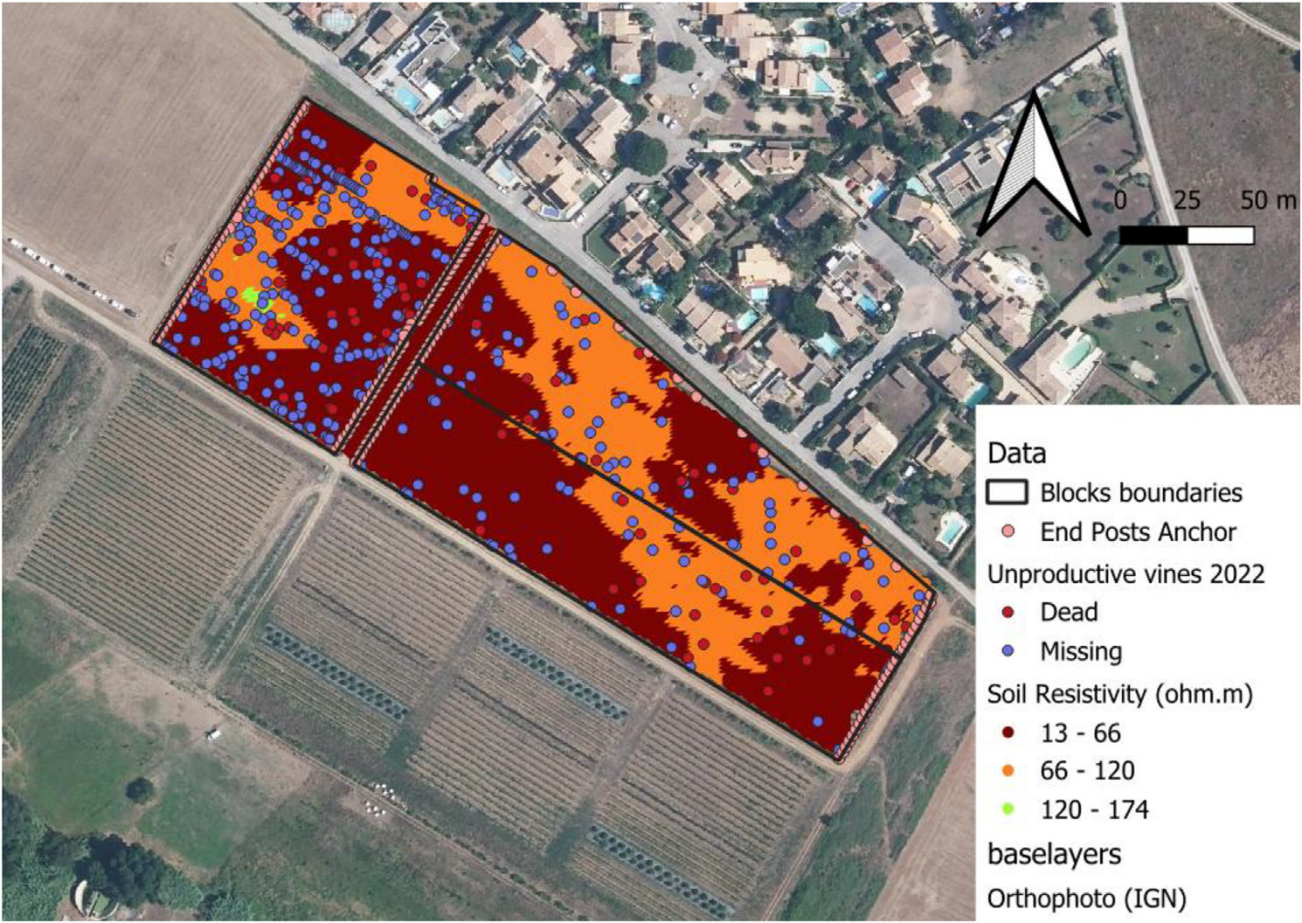
Presentation of the dataset on three blocks of the estate (Base map: Orthophoto from BD ORTHO® of IGN – French National Institute of Geography).

Soil apparent resistivity data (Fig. 2) were acquired by a service provider (Geocarta, Paris, France) with a Multi-depth continuous electrical profiling (MuCep). The MuCep was towed to a geolocated ATV, it sends out an electrical current and measures the soil apparent resistivity. The distance between the emitting probes and the receiving probes allows resistivity to be measured at three depths: 0.5m, 1m and 1.7m. Soil apparent resistivity data were acquired in 2017 and were only available for 37 of the 50 blocks.

**Fig. 3:**
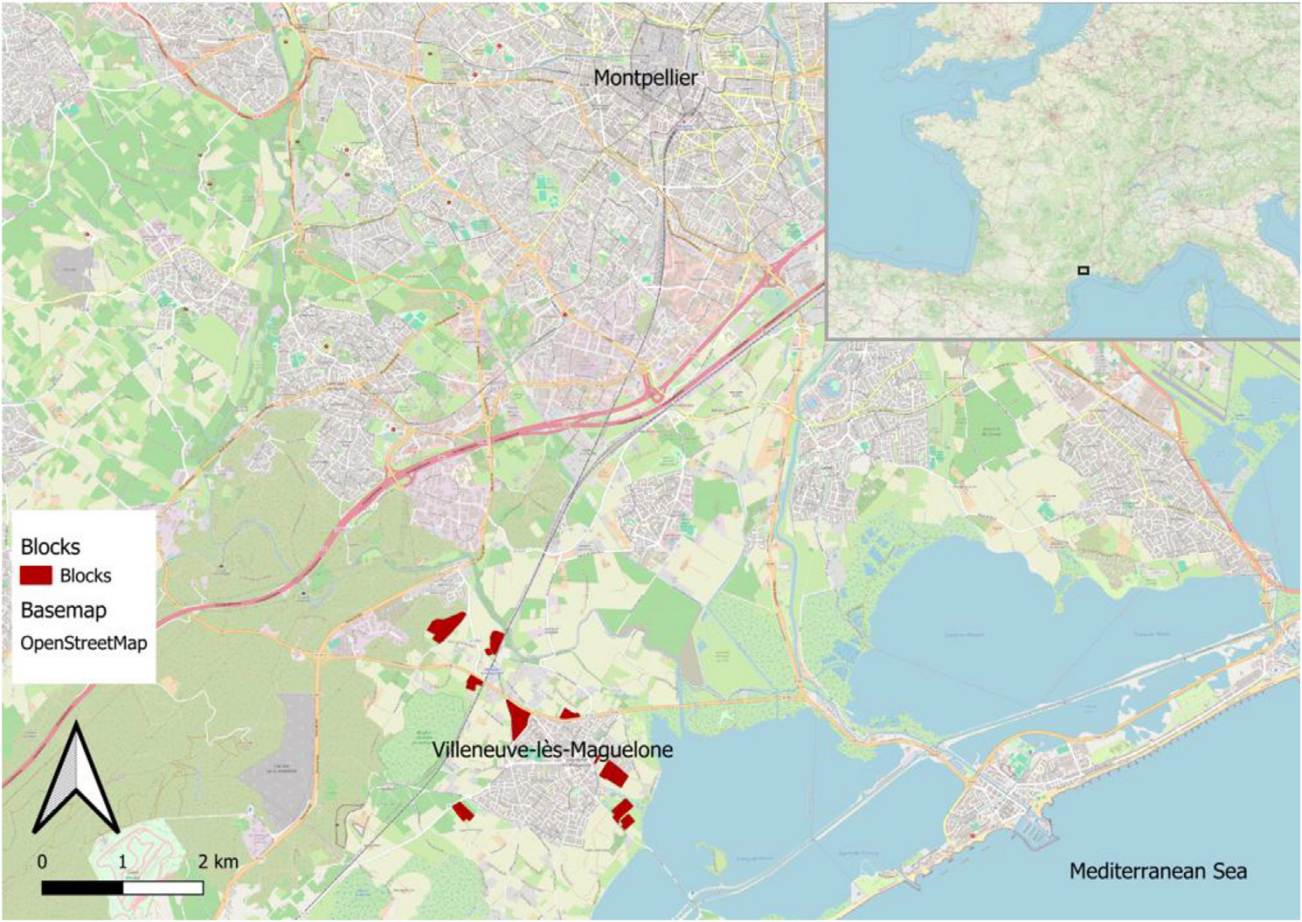
Localisation of the experimental site near Montpellier (Top right: Location of the experimental site in France).

## Limitations

The primary limitation of our data comes from the imprecision inherent in field observations. Even though a strict procedure has been defined to characterise what constitutes a 'dead vine' and a 'missing vine,' real-world interpretation on the ground introduced some uncertainties. For example, judging a dead vine can sometimes be debatable, especially for particularly stunted or severely diseased plants. In addition, the mortality of a plant was estimated by the absence of vegetation at the time when the plant was supposed to have vegetation. However, it is possible that this absence was temporary and that the plant would therefore no longer be classified as dead during a few days after. Another source of imprecision may occur when multiple consecutive vines are absent in a row; in such cases, errors can stem from trying to assess the number of missing vines within a large gap. Both these scenarios inherently lead to some data uncertainties. It's worth noting, however, that repeat counts were implemented with the operator to quantify these sources of error. On the sites chosen for these repetitions, no errors were observed between counts, indicating that if this error exists, it remains very small.

To our knowledge, there is currently no other dataset as exhaustive in cataloguing missing and dead vines in viticulture. However, this dataset is quite localized, representing only a single French wine region with its unique climate, pedological context, and grape varieties. While considerable, the insights that could be generated from this dataset are confined to this specific zone. Consequently, validating this knowledge at different scales will necessitate mobilizing additional data from other regions (in France or in the world).

Finally, the authors acknowledge that this dataset was acquired in a specific production context: Mediterranean climate, large row vineyards (density 4,000 vines/Ha), fully mechanised operations, including mechanical weeding between rows and under rows. The data observed are therefore specific to this particular context. Observations could have presented different characteristics in other contexts. Alone, this dataset cannot provide generic knowledge about vine mortality, the factors that influence it, and its spatial and temporal distribution. Consequently, further data of this type are required across a broader range of technical and pedo-climatic contexts to generate knowledge on vine mortality that can be generalized.

The scope of the data also has its limitations. In order to study vine mortality, there is still some information missing: the causes of mortality (mechanical injury, wood disease, etc.), more information on soil composition and the dynamics of mortality over several years. The climate data corresponding to the experimental context are, of course, important. They are not made available in this study but can be easily downloaded, as documented by [7].

## Ethics Statement

The authors confirm that they have read and follow the ethical requirements for publication in Data in Brief and confirm that the current work does not involve human subjects, animal experiments, or any data collected from social media platforms.

## CRediT Author Statement

**Yoann Valloo**: Conceptualization, Methodology, Investigation, Data Curation, Writing - Original Draft; **Romain Girardot**: Conceptualization, Methodology, Writing - Review & Editing, Project administration, Funding acquisition; **Pauline Faure**: Methodology, Investigation; **Bruno Tisseyre**: Conceptualization, Writing - Review & Editing, Supervision, Funding acquisition

## Data Availability

ZenodoA multi-year dataset of georeferenced unproductive vines in a Mediterranean vineyard (Original data).
